# The extra burden: differential somatization-moderated mediation models of anxiety, depression, and insomnia on quality of life through abnormal illness behavior in Chinese college students

**DOI:** 10.1186/s40359-026-04461-1

**Published:** 2026-04-18

**Authors:** Liyun Liu, Mingqing Huang, Xiaoyun Liu, Sen Yang, Ping Wu, Zhen Pei, Yanqi Wang, Jialiang Mao, Yibo Wang, Junwu Hu, Long Wang

**Affiliations:** 1 Administrative Department of Standardized Training for Resident Doctors, Heping Hospital Affiliated to Changzhi Medical College, Changzhi, 046000 China; 2 Department of Information Management, Heping Hospital Affiliated to Changzhi Medical College, Changzhi, 046000 China; 3 Department of General Practice, The People’s Hospital of Changzhi City, Changzhi, 046000 China; 4 Department of Osteology, The People’s Hospital of Changzhi City, Changzhi, 046000 China; 5 Department of Scientific Research, Heping Hospital Affiliated to Changzhi Medical College, Changzhi, 046000 China; 6 Department of Student Affairs, Changzhi Medical College, Changzhi, 046000 China; 7 Department of Cardiology, Renji Hospital, School of Medicine, Shanghai Jiaotong University, Shanghai, 200000 China; 8 Department of Cardiology, Shanghai Second People’s Hospital, Shanghai, 200011 China; 9 Department of Psychology, School of Public Health, Southern Medical University, Guangzhou, 510515 China; 10 Department of Neurosurgery, Heping Hospital Affiliated to Changzhi Medical College, Changzhi, 046000 China

**Keywords:** Psychological distress, Abnormal illness behavior, Somatization, Quality of life, Moderated mediation model

## Abstract

**Background:**

Psychological distress (anxiety, depression, insomnia) is prevalent among college students, often co-occurring with somatization and abnormal illness behavior (AIB) to impair quality of life (QoL). This study aims to construct moderated mediation models exploring the association between psychological distress and QoL through AIB, and examine the moderating role of somatization, providing evidence for targeted mental health interventions.

**Methods:**

A cross-sectional survey of 7,529 Chinese college students was conducted. Anxiety, depression, and insomnia were treated as predictors, AIB as the mediator, somatization as the moderator, and QoL (mental component summary [MCS] and physical component summary [PCS]) as the outcomes.

**Results:**

After adjusting for selected covariates, anxiety, depression, and insomnia showed significant negative direct effects on PCS (β = −0.0553, 95%CI: −0.0995 to −0.0111; β = −0.1034, 95%CI: −0.1428 to −0.0641; β = −0.3559, 95%CI: −0.3932 to −0.3185) and MCS (β = −0.5321, 95%CI: −0.5819 to −0.4823; β = −0.4799, 95%CI: −0.5243 to −0.4355; β = −0.4818, 95%CI: −0.5247 to −0.4389), all p < 0.05. AIB significantly mediated the associations of anxiety, depression, and insomnia with PCS and MCS. Somatization moderated all indirect effects: conditional indirect effects were significantly negative at low, moderate, and high levels of somatization, and the negative mediating effects strengthened as somatization increased.

**Conclusions:**

AIB mediated the relationship between psychological distress and QoL, and this mediation was moderated by somatization. These findings clarify the psychosomatic correlates of QoL impairment in a non-Western population, informing culturally tailored college mental health strategies and providing implications for clinical assessment and intervention.

**Supplementary Information:**

The online version contains supplementary material available at 10.1186/s40359-026-04461-1.

## Introduction

College students represent a globally prioritized mental health population, primarily due to alarmingly high rates of psychological distress. Internationally, epidemiological studies report that 39.0% of college students experience anxiety, 33.6% suffer from depression [1], and 33.0% have insomnia [2], —symptoms strongly correlating with reduced health-related quality of life (QoL). In China, the National Mental Health Development Report has continuously monitored the mental health status of Chinese college students for 8 years; its 2023–2024 edition, based on a study involving 60,782 Chinese college students, also identified a high prevalence of psychological distress: 44.6% for anxiety and 18.7% for depression, accompanied by clinically significant health-related quality of life impairment [3]. Although insomnia data were not reported in this cohort, the report noted that the risks of depression and anxiety decreased with improved sleep quality. While this association between psychological distress and QoL is consistent across cultures, the specific pathways may differ. In Western populations, psychological distress is more commonly expressed through emotional or affective symptoms, with relatively direct reporting of mood disturbances. In non-Western contexts, by contrast, collectivism and high levels of mental health stigma often lead to the suppression of emotional expression and the manifestation of distress as somatic symptoms [4–8]. This difference in expression pathways suggests that the mechanism linking psychological distress to quality of life may vary between Chinese and Western populations, a pattern supported by classic and contemporary cross-cultural studies on cultural variations in somatic symptom expression [9, 10].

Against this backdrop, two understudied constructs, somatization and abnormal illness behavior (AIB), are critical to unpacking the psychological distress-QoL pathway. From a psychosomatic perspective, psychological distress is associated with elevated somatization and maladaptive cognitive–behavioral responses to health symptoms, which are in turn linked to the development of AIB and poorer health-related quality of life [11, 12]. AIB is defined as the persistence of a maladaptive mode of experiencing, perceiving, evaluating, and responding to one’s own health status, despite clinicians providing a lucid and accurate appraisal of the situation and corresponding management strategies, with sufficient opportunities for discussion, negotiation, and clarification based on a comprehensive assessment of all relevant biological, psychological, social and cultural factors [13–15]. Existing studies have well-documented the significant associations between AIB, psychological distress and adverse health outcomes [16–20]. In addition, somatization, characterized by functional somatic symptoms (i.e., physical symptoms without identifiable organic etiology), is also a well-established correlate of both psychological distress and AIB [21–24]. A study conducted in Japanese general medicine clinics demonstrated that the illness behavior of patients presenting with both functional somatic symptoms and psychological distress was comparable to that of individuals diagnosed with hypochondriasis or major depressive disorder [25]. In contrast, the illness behavior of patients with functional somatic symptoms but no psychological distress closely resembled that of patients whose physical symptoms were attributed to organic diseases [25]. Collectively, these findings underscore that somatization and psychological distress synergistically contribute to the development of AIB. Despite the established theoretical and empirical links among psychological distress, somatization, AIB, and QoL, few studies have simultaneously explored their interactive effects in a unified moderated mediation model. In particular, it remains unclear whether AIB serves as a key mediating pathway underlying the link between psychological distress and reduced QoL, and whether somatization acts as a vulnerability factor that moderates the strength of this pathway. Such a model would help clarify the complex psychosomatic correlates underlying reduced QoL in young adults, especially in non-Western cultural contexts.

To address these limitations, we aim to examine the interrelations between psychological distress, AIB, somatization, and QoL in a large sample of Chinese college students. Specifically, we seek to: (1) Examine the separate associations of anxiety, depression, and insomnia with the physical component summary (PCS) and mental component summary (MCS) of QoL, respectively; (2) Test whether AIB serves as a mediating variable in the relationships between each psychological distress symptom (anxiety, depression, insomnia) and QoL (PCS/MCS); and (3) Investigate whether somatization acts as a moderator that shapes the indirect effect of psychological distress on QoL via AIB. By verifying this moderated mediation model, the present study seeks to clarify the sequential and interactive pathways linking psychological distress, somatization, AIB, and QoL. By clarifying these pathways, this work could provide empirical evidence for the development of culturally tailored interventions to mitigate QoL impairment in college students. Given the high prevalence of psychological distress and somatization among Chinese youth, the findings of this study may hold broader implications for similar cultural contexts globally. 

## Methods

### Study design, participants, and procedure

This was a cross-sectional study conducted among college students in China, with participants specifically recruited from Changzhi Medical College and Changzhi College—two institutions with a national enrollment scope. Data were collected via an online questionnaire survey, which was organized and distributed by the Department of Student Affairs of the respective colleges between April 1 and 14, 2024.

**Fig. 1:**
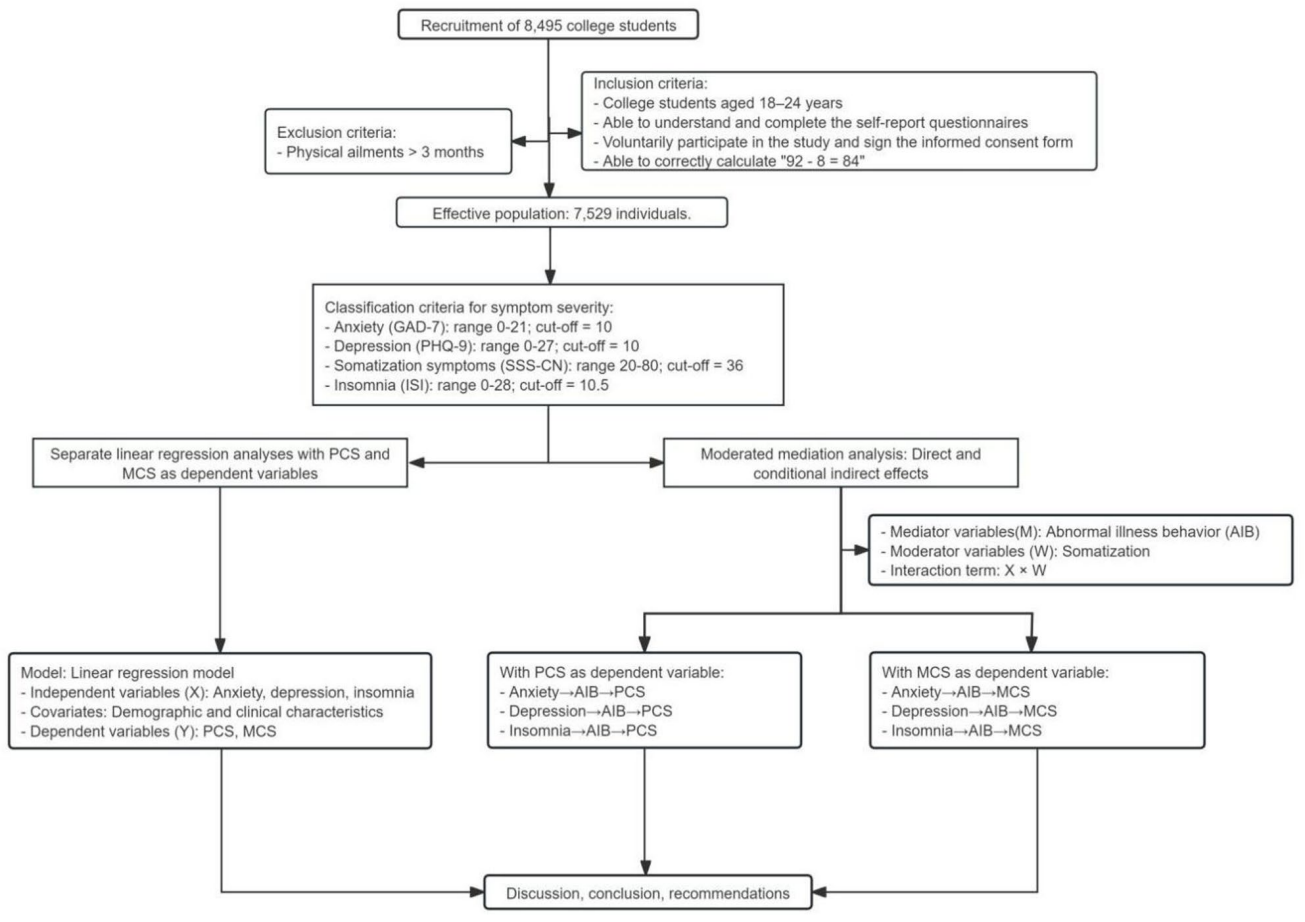
Study Flow Chart

The inclusion criteria were as follows: (1) being a college student aged 18–24 years; (2) having the ability to understand and complete self-reported questionnaires; (3) voluntarily agreeing to participate and signing the informed consent form; and (4) correctly solving the arithmetic problem “92 − 8 = 84” (to verify basic cognitive competence and a serious attitude required for reliable questionnaire completion). The sole exclusion criterion was the presence of physical ailments lasting for ≥ 3 months. Figure 1 illustrates the study flowchart.

### Sample size

Sample size was determined using PASS 15 software based on the prevalence rates of key variables reported in previous studies: 44.6% for anxiety, 18.7% for depression [3], 20.4 to 25.5% for insomnia [26, 27], and 34.85% for somatization [28]. Anxiety exhibited the highest prevalence (44.6%) among these variables. A two-sided test with a significance level of 0.05 and a margin of error of 0.02 yielded a required sample size of 2,421 cases for this highest prevalence rate. To account for a potential 50% invalid response rate due to the use of an extensive questionnaire, the final required sample size was adjusted to 4,842 cases.

### Measures

#### Outcome variables (Dependent variables)

Quality of life: Assessed by the Short-Form Health Survey 12 (SF-12). It is a 12-item questionnaire tool for health outcomes, covering general health (GH), physical functioning (PF), bodily pain (BP), vitality (VT), mental health (MH), social functioning (SF), and the limitations imposed by physical roles (RP) and emotional roles (RE). The single-item measures are scored so that high scores indicate better functioning and higher levels of pain. All scores are transformed linearly to 0-100 scales, with 0 representing the lowest and 100 the highest possible scores [29]. Based on these eight domain scores, the physical component summary (PCS) (reflecting physical health-related quality of life) and mental component summary (MCS) (reflecting mental health-related quality of life) were calculated using standard algorithms [30]. The formulas are as follows:

$$\begin{aligned} &\mathrm{PCS}=\left[\left(\mathrm{GHZ}\ast{0.24954}\right)+\left(\mathrm{PFZ}\ast{0.42402}\right)+\left(\mathrm{RPZ}\ast{0.35119}\right)\right. \\& \quad\quad\quad \left.+\left(\mathrm{REZ}\ast{-0.19206}\right) +\left(\mathrm{BPZ}\ast{0.31754}\right)+\left(\mathrm{MHZ}\ast{-0.22069}\right)\right. \\& \quad\quad\quad \left.+\left(\mathrm{VTZ}\ast{0.02877}\right)+\left(\mathrm{SFZ}\ast{-0.00753}\right)\right]\ast{10+50};\;\\&\mathrm{MCS}=\left[\left(\mathrm{GHZ}\ast{-0.01571}\right)+\left(\mathrm{PFZ}\ast{-0.22999}\right)+\left(\mathrm{RPZ}\ast{-0.12329}\right) \right. \\& \quad\quad\quad \left.+\left(\mathrm{REZ}\ast{0.43407}\right)+\left(\mathrm{BPZ}\ast{-0.09731}\right)+\left(\mathrm{MHZ}\ast{0.48581}\right)\right. \\& \quad\quad\quad \left.+\left(\mathrm{VTZ}\ast{0.23534}\right) +\left(\mathrm{SFZ}\ast{0.26876}\right)\right]\ast{10+50} \end{aligned}$$

#### Predictors (Independent variables)

Psychological distress was measured using three validated scales:

- *Anxiety*: Assessed by the Generalized Anxiety Scale-7 (GAD-7). Each item is rated on a 4-point Likert scale (0 = not at all to 3 = nearly every day), with total scores ranging from 0 to 21. A score ≥ 10 indicates clinically significant anxiety [31–33]. The standardized McDonald’s omega was 0.932 in this study.
- *Depression*: Assessed by the Patient Health Questionnaire-9 (PHQ-9). Each item is rated on a 4-point Likert scale (0 = not at all to 3 = nearly every day), with total scores ranging from 0 to 27. A score ≥ 10 indicates clinically significant depression [34]. The standardized McDonald’s omega was 0.914 in this study.
- *Insomnia*: Assessed by the Insomnia Severity Index (ISI). It consists of 7 items rated on a 5-point Likert scale (0 = no problem to 4 = very severe problem), with total scores ranging from 0 to 28. A score ≥ 10.5 indicates clinically significant insomnia [35–38]. The standardized McDonald’s omega was 0.877 in this study.

#### Mediator

Abnormal Illness Behavior (AIB): Assessed by the AIB domain of the Psychosocial Index-Young (PSI-Y). This domain consists of 3 items rated on a 4-point Likert scale (0 = never to 3 = most of the time), with total scores ranging from 0 to 9 [39, 40]. The standardized McDonald’s omega was 0.857 in this study. 

#### Moderator

Somatization: Assessed by the Somatization Self-Rating Scale-China (SSS-CN). It consists of 20 items rated on a 4-point Likert scale (1 = normal to 4 = severe), with total scores ranging from 20 to 80. A score ≥ 36 indicates clinically significant somatization [41–45]. The standardized McDonald’s omega was 0.948 in this study.

#### Covariates

Covariates were primarily selected based on the Psychosocial Index-Young (PSI-Y). It consists of 12 items related to socio-demographic and clinical data, with an additional 39 items encompassing five domains. In the present study, socio-demographic and clinical data included: daily study duration (categorized as < 6 h vs. ≥ 6 h); medical history (chronic physical ailments lasting ≥ 3 months, mental illness, hospitalization, and allergy); medication use; and lifestyle factors (cigarette smoking, alcohol drinking, coffee/tea consumption). Since recreational drug use is illegal in China, this variable was replaced with daily exercise duration (categorized as < 0.5 h vs. ≥0.5 h). The five domains included stress (items 12–26), well-being (items 27–32), psychological distress (items 33–47), abnormal illness behavior (items 48–50), and quality of life (item 51) [39, 40]. McDonald’s omega values for the domains were: stress (0.683), and psychological distress (0.943). Well-being, which includes three subdimensions, was not suitable for McDonald’s omega calculation.

Additional variables were supplemented via ad hoc questions, including age, gender, academic classification (categorized as medical vs. non-medical students), geographical region of origin (East/Central/West/Northeast China), family relations status, and multidimensional health perception (including bio-psycho-social factors, lifestyle, environment, and medical conditions).

### Moderated mediation model design

**Fig. 2:**
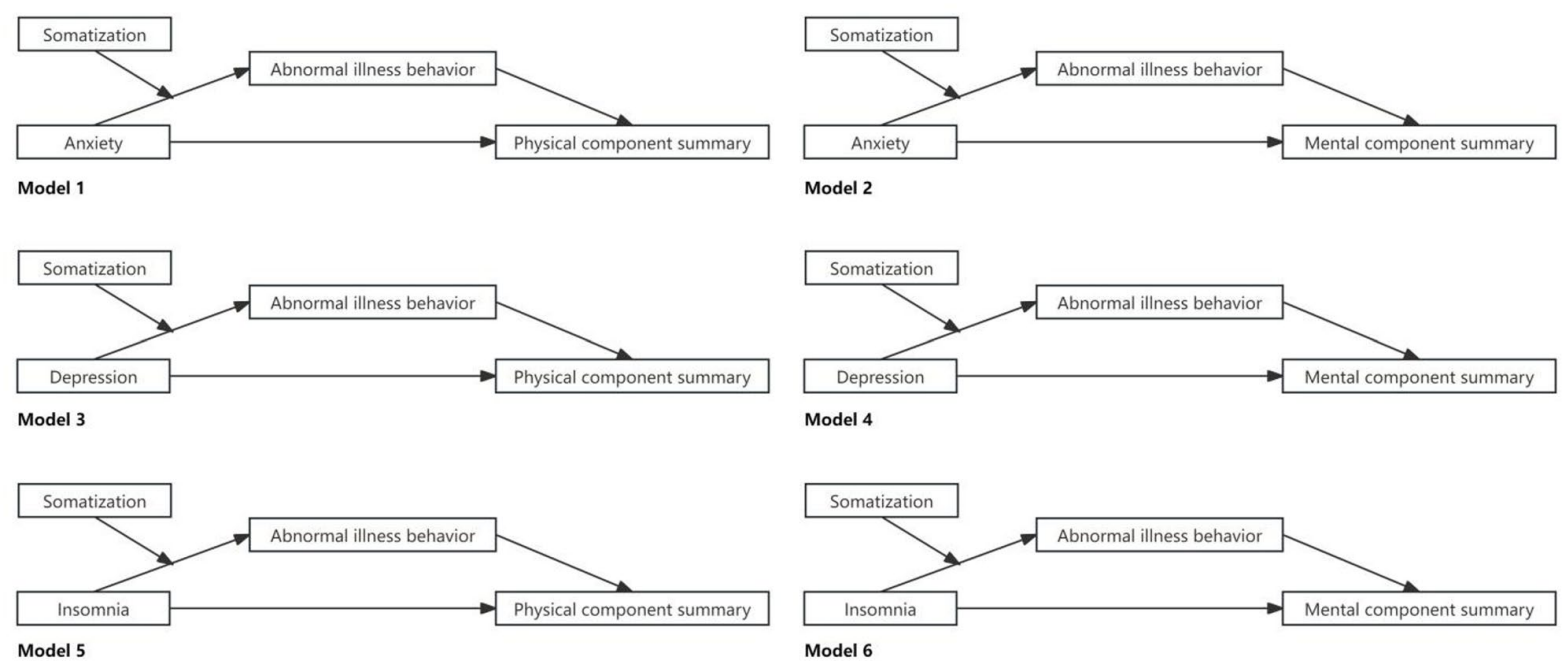
Schematic diagram of the moderated mediation model: Anxiety /Depression / Insomnia → Abnormal illness behavior (AIB) → Physical Component Summary (PCS) / Mental Component Summary (MCS), with somatization as the moderator variable

Figure 2 presents the conceptual framework of the six moderated mediation models tested in this study. Each model examines the mediating role of AIB in the association between a psychological distress variable (anxiety, depression, or insomnia) and a QoL dimension (PCS or MCS), with somatization serving as the moderator of the indirect path. Specifically: Models 1, 3, and 5 focus on PCS as the dependent variable (paired with anxiety, depression, and insomnia, respectively); Models 2, 4, and 6 focus on MCS as the dependent variable (paired with anxiety, depression, and insomnia, respectively). Multicollinearity diagnostics were conducted and no severe multicollinearity was identified. Given the high conceptual and empirical overlap among anxiety, depression, and insomnia, these psychological distress indicators were entered into separate regression models to prevent estimation bias and ensure robust results.

### Statistical analyses

All statistical analyses were performed using SPSS 26.0 and the PROCESS Macro 4.1 for moderated mediation analysis. The significance level was set at α = 0.05 (two-tailed).

Descriptive statistics: Continuous variables were reported as mean ± standard deviation (SD) or median (interquartile range, IQR) based on the Shapiro-Wilk test for normality. Categorical variables were reported as frequencies and percentages.

Correlational analysis: Spearman’s rank correlation coefficients were used to examine bivariate associations among key variables due to the non-normal distribution of continuous variables.

Multicollinearity was diagnosed using the variance inflation factor (VIF), condition index and variance proportions. Diagnostic criteria were defined as follows: severe multicollinearity if VIF ≥ 10, significant multicollinearity risk if condition index ≥ 30, and information redundancy among variables if variance proportions ≥ 0.5 for multiple variables under the same condition index dimension.

Moderated mediation analysis: PROCESS Macro 4.1 Model 7 was used to test the moderated mediation model. The model examined: (1) whether AIB mediates the associations between psychological distress (anxiety/depression/insomnia) and QoL (PCS/MCS); (2) whether somatization moderates the indirect effect of psychological distress on QoL via AIB. Bootstrap resampling (5,000 iterations) was used to calculate 95% confidence intervals (CIs) for direct effects, conditional indirect effects and the index of moderated mediation. A CI excluding zero indicates a significant effect. All continuous variables were mean-centered to simplify the interpretation of moderating effects and reduce potential multicollinearity among independent variables (including interaction terms). All selected covariates were included in the moderated mediation models to adjust for potential confounding.

## Results

A total of 8,495 college students were initially invited to participate; after applying the predefined inclusion and exclusion criteria, 7,529 valid responses were retained, corresponding to an effective response rate of 88.6%.

### Descriptive statistics of key variables

Table 1 summarizes the descriptive statistics, ranges, and clinically relevant proportions (where applicable) of primary outcomes, independent variables, mediator, moderator, and key covariates. The PCS and MCS had mean ± SD of 49.216 ± 5.938 and 51.055 ± 7.791, respectively. For psychological distress indicators (independent variables), anxiety, depression, and insomnia were reported as median (IQR): 2 (0, 6), 3 (0, 7), and 3 (1, 6), with total risk proportions of 32.5% (*n* = 2448), 37.3% (*n* = 2805), and 18.3% (*n* = 1381), and clinically relevant proportions of 4.7% (*n* = 352), 6.8% (*n* = 512), and 7.6% (*n* = 570), respectively. The mediator AIB had a median (IQR) of 0 (0, 1). The moderator somatization showed a median (IQR) of 23 (20, 29), with 12.3% (*n* = 924) meeting the clinically relevant threshold (≥ 36). Regarding covariates, the sample included 51.0% medical students (*n* = 3843), 74.9% females (*n* = 5640), and had a median (IQR) age of 20 (19, 21) years. Most participants were from central China (82.1%, *n* = 6183). Over half of students reported daily study duration < 6 h (56.0% ), and 63.4% engaged in ≥ 0.5 h of daily exercise. History of mental illness, hospitalization, allergies, and regular medication use were reported in 3.8%, 18.1%, 11.8%, and 0.6% of participants, respectively. The PSI stress and well-being domains had median (IQR) of 3 (2, 4) and 5 (4, 6).

**Table 1:** Descriptive statistics of study variables

Variables	Summary statistic	Range	Total risk proportion (*n*, %)	Clinically relevant proportion (*n*, %)
**Primary Outcome (Y)**				
Physical component summary (PCS)	49.216 ± 5.938			
Mental component summary (MCS)	51.055 ± 7.791			
**Independent Variables (X)**				
Anxiety (GAD-7)	2(0,6)	0–21	≥ 5: 2448(32.5)	≥ 10: 352(4.7)
Depression (PHQ-9)	3(0,7)	0–37	≥ 5: 2805(37.3)	≥ 10: 512(6.8)
Insomnia (ISI)	3(1,6)	0–28	≥ 8: 1381(18.3)	≥ 10.5: 570(7.6)
**Mediator (M)**				
Abnormal illness behavior (AIB)	0(0,1)	0–9		-
Perceived medical misdiagnosis	0(0,0)	0–3		-
Hypochondriasis	0(0,1)	0–3		-
Heightened bodily sensation	0(0,1)	0–3		-
**Moderator (W)**				
Somatization (SSS-CN)	23(20,29)	20–80	≥ 30: 1729(23.0)	≥ 36: 924(12.3)
**Key Covariates**				
Professional classification				
Medical students	3843(51)	-		-
Non-medical students	3686(49)	-		-
Gender				
Male	1889(25.1)	-		-
Female	5640(74.9)	-		-
Age	20(19,21)	18–24		
Geographical regions				
East	426(5.7)	-		-
Central	6183(82.1)	-		-
West	757(10.1)	-		-
Northeast	163(2.2)	-		-
Study duration				
<6 h	4217(56)	-		-
≥6 h	3312(44)	-		-
Exercise duration				
<0.5 h	2754(36.6)	-		-
≥0.5 h	4775(63.4)	-		-
Medical History				
Mental illness	284(3.8)	-		-
Hospitalization	1360(18.1)	-		-
Allergies	890(11.8)	-		-
Medication use	43(0.6)	-		-
Lifestyle factors				
Cigarette smoking	144(1.9)	-		-
Alcohol drinking	117(1.6)	-		-
Coffee or tea consumption	1526(20.3)	-		-
Harmonious family relations	7175(95.3)	-		-
Multidimensional health perception	3079(40.9)	-		-
PSI domains				
Stress	3(2,4)	0–15		-
Well-being	5(4,6)	0–6		-

Continuous normally distributed variables are presented as mean ± SD, continuous skewed variables as median (Q1, Q3), categorical variables as n (%), and clinically relevant proportions refer to the proportion of participants with scores above the cut-off values of each scale. For the 16th, 50th, and 84th percentiles of somatization (a non-normal variable), the calculated values are 20, 23, and 33, respectively

### Correlational analyses among key variables

Table 2 presents Spearman’s rank correlation coefficients among key variables in the moderated mediation model. All psychological distress indicators (anxiety, depression, insomnia) showed significant positive correlations with the mediator AIB (*r* = 0.495, 0.533, 0.439, respectively; all *p* < 0.01) and the moderator somatization (*r* = 0.696, 0.764, 0.540, respectively; all *p* < 0.01), with depression and somatization exhibiting the strongest association. For quality of life dimensions (PCS and MCS), significant negative correlations were observed with anxiety, depression, insomnia, AIB, and somatization (all *p* < 0.01). Notably, insomnia was most strongly associated with lower PCS (*r* = -0.316) and also showed a prominent negative association with MCS (*r* = -0.450), followed by anxiety and depression (*r* = -0.419 and − 0.426 for MCS, respectively). AIB was moderately negatively correlated with both PCS (*r* = -0.234) and MCS (*r* = -0.393), supporting its potential role as a mediator. Additionally, somatization showed moderate to strong positive correlations with all psychological distress indicators and AIB (*r* = 0.439–0.764), which is a prerequisite for its proposed moderating effect. A weak but significant positive correlation was found between PCS and MCS (*r* = 0.072, *p* < 0.01). These correlational patterns provide initial support for the proposed moderated mediation framework, justifying subsequent formal testing of AIB’s mediating role and somatization’s moderating role.

**Table 2:** Correlation analysis of key variables

Variable	1	2	3	4	5	6	7
1.Anxiety	1						
2.Depression	0.806**	1					
3.Insomnia	0.486**	0.544**	1				
4.Abnormal illness behavior (AIB)	0.495**	0.533**	0.439**	1			
5.Somatization	0.696**	0.764**	0.540**	0.576**	1		
6.Physical component summary (PCS)	− 0.180**	− 0.203**	− 0.316**	− 0.234**	− 0.223**	1	
7.Mental component summary (MCS)	− 0.419**	− 0.426**	− 0.450**	− 0.393**	− 0.383**	0.072**	1

** indicates *P* < 0.01 in Spearman’s rank correlation analysis, two-tailed test

### Selection of covariates: univariate linear regression with PCS/MCS as dependent variables

Univariate analysis was performed using linear regression within the generalized linear model, with PCS/MCS as the dependent variable. Among these significant covariates, non-medical students, history of mental illness, history of medication use, history of hospitalization, alcohol drinking, coffee or tea consumption, and total stress were negatively associated with PCS; study duration ≥ 6 h, exercise duration ≥ 0.5 h, multidimensional health perception and well-being were positively associated with PCS (Supplementary Table 1). Additionally, non-medical students, age, history of mental illness, history of medication use, history of hospitalization, alcohol drinking, cigarette smoking, coffee or tea consumption, and total stress were negatively associated with MCS, while study duration ≥ 6 h, exercise duration ≥ 0.5 h, multidimensional health perception and well-being were positively associated with MCS (Supplementary Table 2). To maintain the consistency of covariates, all the above covariates were selected for multicollinearity diagnosis and moderated mediation effect analysis in each model.

### Multicollinearity diagnosis: multiple linear regression analysis with PCS/MCS as the dependent variables

Supplementary Tables 3 and 4 present the results of multiple linear regression analyses examining the associations of psychological distress indicators (anxiety, depression, insomnia) and selected covariates with PCS and MCS, respectively, with the selected variables adjusted for in each model. Combined indicators for multicollinearity diagnosis: the VIF value < 10, the maximum condition index (CI) < 30, and the variance proportions of multiple independent variables under any CI dimension ≤ 0.5. There was no severe multicollinearity in any of the models, and the regression coefficient estimates were stable and reliable. All models showed good overall fit, and all p-values < 0.001, indicating that the models were statistically significant.

### Somatization-moderated mediation effects

Moderated mediation analysis was used to examine whether AIB mediates the associations of anxiety, depression, and insomnia with quality of life (PCS and MCS), and whether this mediating effect is moderated by somatization. The 16th, 50th, and 84th percentiles represent low, moderate, and high somatization levels, respectively, with corresponding SSS-CN scores of 20, 23, and 33.

Table 3 presents the moderated mediation effect analysis without covariates. The results showed that AIB significantly mediated the relationships of anxiety, depression, and insomnia with PCS and MCS, and these mediating effects were moderated by somatization. All conditional indirect effects and the moderated mediation index were statistically significant.

**Table 3:** Moderated mediation effect analysis without covariates

Model	Effect type	Effect (B)	Standard error	t	95% Confidence interval	*P*
(Predictor→Mediator→Outcome)						
Anxiety→AIB→PCS	Direct effect (Anxiety→PCS)	-0.1178	0.0210	-5.6043	(-0.1591,-0.0766)	< 0.001
	Conditional indirect effect (Anxiety→AIB→PCS)					
	Low somatization (16%=-5.8651)	-0.0309	0.0066	-	(-0.0445,-0.0182)*	< 0.05
	Moderate somatization (50%=-2.8651)	-0.0335	0.0064	-	(-0.0465,-0.0210)*	< 0.05
	High somatization (84%=7.1349)	-0.0420	0.0077	-	(-0.0577,-0.0271)*	< 0.05
	Index of moderated mediation (Somatization as moderator)	-0.0009	0.0005	-	(-0.0018,0)*	< 0.05
Anxiety→AIB→MCS	Direct effect (Anxiety→MCS)	-0.7305	0.0248	-29.4612	(-0.7791,-0.6819)	< 0.001
	Conditional indirect effect (Anxiety→AIB→MCS)					
	Low somatization (16%=-5.8651)	-0.0431	0.0090	-	(-0.0613,-0.0263)*	< 0.05
	Moderate somatization (50%=-2.8651)	-0.0467	0.0087	-	(-0.0642,-0.0305)*	< 0.05
	High somatization (84%=7.1349)	-0.0586	0.0103	-	(-0.0798,-0.0394)*	< 0.05
	Index of moderated mediation (Somatization as moderator)	-0.0012	0.0006	-	(-0.0025,0)*	< 0.05
Depression→AIB→PCS	Direct effect (Depression→PCS)	-0.1509	0.0187	-8.0577	(-0.1877,-0.1142)	< 0.001
	Conditional indirect effect (Depression→AIB→PCS)					
	Low somatization (16%=-5.8651)	-0.0317	0.0058	-	(-0.0435,-0.0206)*	< 0.05
	Moderate somatization (50%=-2.8651)	-0.0341	0.0058	-	(-0.0459,-0.0232)*	< 0.05
	High somatization (84%=7.1349)	-0.0421	0.0070	-	(-0.0563,-0.0287)*	< 0.05
	Index of moderated mediation (Somatization as moderator)	-0.0008	0.0003	-	(-0.0015,-0.0001)*	< 0.05
Depression→AIB→MCS	Direct effect (Depression→MCS)	-0.6661	0.0221	-30.1606	(-0.7093,-0.6228)	< 0.001
	Conditional indirect effect (Depression→AIB→MCS)					
	Low somatization (16%=-5.8651)	-0.0440	0.0079	-	(-0.0594,-0.0285)*	< 0.05
	Moderate somatization (50%=-2.8651)	-0.0473	0.0078	-	(-0.0626,-0.0320)*	< 0.05
	High somatization (84%=7.1349)	-0.0584	0.0093	-	(-0.0771,-0.0405)*	< 0.05
	Index of moderated mediation (Somatization as moderator)	-0.0011	0.0005	-	(-0.0021,-0.0002)*	< 0.05
Insomnia→AIB→PCS	Direct effect (Insomnia→PCS)	-0.3769	0.0182	-20.6715	(-0.4127,-0.3412)	< 0.001
	Conditional indirect effect (Insomnia→AIB→PCS)					
	Low somatization (16%=-5.8651)	-0.0282	0.0044	-	(-0.0374,-0.0201)*	< 0.05
	Moderate somatization (50%=-2.8651)	-0.0300	0.0043	-	(-0.0389,-0.0220)*	< 0.05
	High somatization (84%=7.1349)	-0.0360	0.0049	-	(-0.0461,-0.0269)*	< 0.05
	Index of moderated mediation (Somatization as moderator)	-0.0006	0.0002	-	(-0.0011,-0.0002)*	< 0.05
Insomnia→AIB→MCS	Direct effect (Insomnia→MCS)	-0.6473	0.0221	-29.3337	(-0.6905,-0.6040)	< 0.001
	Conditional indirect effect (Insomnia→AIB→MCS)					
	Low somatization (16%=-5.8651)	-0.0677	0.0084	-	(-0.0844,-0.0516)*	< 0.05
	Moderate somatization (50%=-2.8651)	-0.0719	0.0079	-	(-0.0879,-0.0567)*	< 0.05
	High somatization (84%=7.1349)	-0.0862	0.0089	-	(-0.1039,-0.0693)*	< 0.05
	Index of moderated mediation (Somatization as moderator)	-0.0014	0.0006	-	(-0.0026,-0.0003)*	< 0.05

All effects are unstandardized coefficients (B); * indicates the 95% Bootstrap confidence intervals (5,000 iterations) exclude 0, denoting statistical significance at α = 0.05. All covariates are included in the moderated mediation models to adjust for potential confounding

Table 4 presents the moderated mediation effect analysis with selected covariates. After adjusting for all selected covariates, the results remained robust and consistent with the unadjusted model.

**Table 4:** Moderated mediation effect analysis with selected covariates

Model	Effect type	Effect (B)	Standard error	t	95% Confidence interval	*P*
(Predictor→Mediator→Outcome)						
Anxiety→AIB→PCS	Direct effect (Anxiety → PCS)	-0.0553	0.0225	-2.4528	(-0.0995,-0.0111)	0.0142
	Conditional indirect effect (Anxiety → AIB → PCS)					
	Low somatization (16%=-5.8651)	-0.0192	0.0061	-	(-0.0320,-0.0078)*	< 0.05
	Moderate somatization (50%=-2.8651)	-0.0220	0.0059	-	(-0.0342,-0.0112)*	< 0.05
	High somatization (84%=7.1349)	-0.0312	0.0070	-	(-0.0454,-0.0178)*	< 0.05
	Index of moderated mediation (Somatization as moderator)	-0.0009	0.0004	-	(-0.0017,-0.0001)*	< 0.05
Anxiety→AIB→MCS	Direct effect (Anxiety → MCS)	-0.5321	0.0254	-20.9380	(-0.5819,-0.4823)	< 0.001
	Conditional indirect effect (Anxiety → AIB → MCS)					
	Low somatization (16%=-5.8651)	-0.0218	0.0071	-	(-0.0357,-0.0081)*	< 0.05
	Moderate somatization (50%=-2.8651)	-0.0249	0.0069	-	(-0.0386,-0.0118)*	< 0.05
	High somatization (84%=7.1349)	-0.0354	0.0080	-	(-0.0513,-0.0202)*	< 0.05
	Index of moderated mediation (Somatization as moderator)	-0.0010	0.0005	-	(-0.0020,-0.0001)*	< 0.05
Depression→AIB→PCS	Direct effect (Depression → PCS)	-0.1034	0.0201	-5.1505	(-0.1428,-0.0641)	< 0.001
	Conditional indirect effect (Depression → AIB → PCS)					
	Low somatization (16%=-5.8651)	-0.0220	0.0054	-	(-0.0328,-0.0114)*	< 0.05
	Moderate somatization (50%=-2.8651)	-0.0244	0.0054	-	(-0.0352,-0.0142)*	< 0.05
	High somatization (84%=7.1349)	-0.0326	0.0064	-	(-0.0457,-0.0208)*	< 0.05
	Index of moderated mediation (Somatization as moderator)	-0.0008	0.0003	-	(-0.0015,-0.0002)*	< 0.05
Depression→AIB→MCS	Direct effect (Depression → MCS)	-0.4799	0.0227	-21.1852	(-0.5243,-0.4355)	< 0.001
	Conditional indirect effect (Depression → AIB → MCS)					
	Low somatization (16%=-5.8651)	-0.0250	0.0063	-	(-0.0380,-0.0134)*	< 0.05
	Moderate somatization (50%=-2.8651)	-0.0277	0.0062	-	(-0.0406,-0.0159)*	< 0.05
	High somatization (84%=7.1349)	-0.0371	0.0073	-	(-0.0518,-0.0233)*	< 0.05
	Index of moderated mediation (Somatization as moderator)	-0.0009	0.0004	-	(-0.0017,-0.0002)*	< 0.05
Insomnia→AIB→PCS	Direct effect (Insomnia → PCS)	-0.3559	0.0191	-18.6791	(-0.3932,-0.3185)	< 0.001
	Conditional indirect effect (Insomnia → AIB → PCS)					
	Low somatization (16%=-5.8651)	-0.0231	0.0043	-	(-0.0324,-0.0153)*	< 0.05
	Moderate somatization (50%=-2.8651)	-0.0251	0.0042	-	(-0.0341,-0.0173)*	< 0.05
	High somatization (84%=7.1349)	-0.0317	0.0048	-	(-0.0415,-0.0227)*	< 0.05
	Index of moderated mediation (Somatization as moderator)	-0.0007	0.0002	-	(-0.0011,-0.0002)*	< 0.05
Insomnia→AIB→MCS	Direct effect (Insomnia → MCS)	-0.4818	0.0219	-21.9998	(-0.5247,-0.4389)	< 0.001
	Conditional indirect effect (Insomnia → AIB → MCS)					
	Low somatization (16%=-5.8651)	-0.0423	0.0063	-	(-0.0554,-0.0302)*	< 0.05
	Moderate somatization (50%=-2.8651)	-0.0459	0.0060	-	(-0.0586,-0.0345)*	< 0.05
	High somatization (84%=7.1349)	-0.0580	0.0068	-	(-0.0727,-0.0455)*	< 0.05
	Index of moderated mediation (Somatization as moderator)	-0.0012	0.0004	-	(-0.0020,-0.0004)*	< 0.05

All effects are unstandardized coefficients (B); * indicates the 95% Bootstrap confidence intervals (5,000 iterations) exclude 0, denoting statistical significance at α = 0.05. All covariates are included in the moderated mediation models to adjust for potential confounding

#### For PCS as the outcome

Anxiety showed a significant negative direct effect on PCS (β = -0.0553, 95% CI: -0.0995 to -0.0111, *p* = 0.0142). The conditional indirect effects via AIB were significantly negative at low (β = -0.0192, 95% CI: -0.0320 to -0.0078), moderate (β = -0.0220, 95% CI: -0.0342 to -0.0112), and high (β = -0.0312, 95% CI: -0.0454 to -0.0178) levels of somatization. The index of moderated mediation was significant (β = -0.0009, 95% CI: -0.0017 to -0.0001), indicating that higher somatization was associated with a stronger negative indirect association between anxiety and PCS through AIB.

Depression showed a significant negative direct effect on PCS (β = -0.1034, 95% CI: -0.1428 to -0.0641, *p* < 0.001). The conditional indirect effects via AIB were significantly negative across low (β = -0.0220, 95% CI: -0.0328 to -0.0114), moderate (β = -0.0244, 95% CI: -0.0352 to -0.0142), and high (β = -0.0326, 95% CI: -0.0457 to -0.0208) somatization levels. The index of moderated mediation was significant (β = -0.0008, 95% CI: -0.0015 to -0.0002), indicating that higher somatization was associated with a stronger negative indirect association between depression and PCS through AIB.

Insomnia showed a significant negative direct effect on PCS (β = -0.3559, 95% CI: -0.3932 to -0.3185, *p* < 0.001). The conditional indirect effects via AIB were significantly negative at low (β = -0.0231, 95% CI: -0.0324 to -0.0153), moderate (β = -0.0251, 95% CI: -0.0341 to -0.0173), and high (β = -0.0317, 95% CI: -0.0415 to -0.0227) somatization levels. The index of moderated mediation was significant (β = -0.0007, 95% CI: -0.0011 to -0.0002), indicating that higher somatization was associated with a stronger negative indirect association between insomnia and PCS through AIB. 

#### For MCS as the outcome

Anxiety showed a significant negative direct effect on MCS (β = -0.5321, 95% CI: -0.5819 to -0.4823, *p* < 0.001). The conditional indirect effects via AIB were significantly negative at low (β = -0.0218, 95% CI: -0.0357 to -0.0081), moderate (β = -0.0249, 95% CI: -0.0386 to -0.0118), and high (β = -0.0354, 95% CI: -0.0513 to -0.0202) somatization levels. The index of moderated mediation was significant (β = -0.0010, 95% CI: -0.0020 to -0.0001), suggesting that higher somatization was associated with a stronger negative indirect association between anxiety and MCS through AIB.

Depression showed a significant negative direct effect on MCS (β = -0.4799, 95% CI: -0.5243 to -0.4355, *p* < 0.001). The conditional indirect effects via AIB were significantly negative across low (β = -0.0250, 95% CI: -0.0380 to -0.0134), moderate (β = -0.0277, 95% CI: -0.0406 to -0.0159), and high (β = -0.0371, 95% CI: -0.0518 to -0.0233) somatization levels. The index of moderated mediation was significant (β = -0.0009, 95% CI: -0.0017 to -0.0002), suggesting that higher somatization was associated with a stronger negative indirect association between depression and MCS through AIB.

Insomnia showed a significant negative direct effect on MCS (β = -0.4818, 95% CI: -0.5247 to -0.4389, *p* < 0.001). The conditional indirect effects via AIB were significantly negative at low (β = -0.0423, 95% CI: -0.0554 to -0.0302), moderate (β = -0.0459, 95% CI: -0.0586 to -0.0345), and high (β = -0.0580, 95% CI: -0.0727 to -0.0455) somatization levels. The index of moderated mediation was significant (β = -0.0012, 95% CI: -0.0020 to -0.0004), suggesting that higher somatization was associated with a stronger negative indirect association between insomnia and MCS through AIB. 

## Discussion

The present cross-sectional study examined the potential associative pathways linking psychological distress (anxiety, depression, insomnia) and QoL in a large cohort of Chinese college students, focusing on the mediating role of AIB and the moderating role of somatization. Three key findings emerged consistently both before and after adjusting for covariates: First, anxiety, depression, and insomnia were each independently and negatively associated with both PCS and MCS. Second, AIB significantly mediated the associations between each type of psychological distress and QoL. Third, somatization moderated these indirect effects, with the mediated pathways becoming stronger at higher levels of somatization. These findings improve understanding of the potential psychosomatic correlates underlying reduced QoL among non-Western college students and highlight the value of an integrated approach that targets somatization and abnormal illness behavior in addition to psychological distress, to more effectively improve physical and mental quality of life in this population.

Consistent with global trends [1, 2] and local evidence in China [3], our findings revealed distinct profiles in the type and severity of psychological distress. While the total risk proportion (subclinical plus clinically relevant cases) for anxiety, depression, and insomnia reached 32.5%, 37.3%, and 18.3%, respectively, clinically relevant cases (moderate-to-severe) were substantially lower at 4.7%, 6.8%, and 7.6%. The discrepancy underscores that psychological distress exists as a continuous spectrum rather than a dichotomous “ill and not ill” classification. This dual-tiered prevalence pattern highlights the importance of characterizing the full symptom spectrum, thereby justifying our focus on the mechanisms by which AIB and somatization modulate the association between psychological distress and QoL impairment in this population. These prevalence estimates also provide practical implications for campus mental health screening, suggesting that universal and spectrum-based monitoring may be more sensitive than a binary case-ascertainment strategy for identifying students at risk of reduced quality of life.

The correlation analysis provides foundational evidence for the interconnectedness of key variables in our conceptual model, aligning with prior psychosomatic research. First, the strong positive correlations among the three psychological distress indicators—anxiety, depression, and insomnia—are consistent with extensive epidemiological findings [46–48]. Anxiety and depression exhibited a very high correlation (*r* = 0.806), reflecting their well-documented comorbidity and shared affective underpinnings [49–52]; meanwhile, both anxiety and depression were moderately correlated with insomnia (*r* = 0.486 and *r* = 0.544, respectively), which supports the associative relationship between mood disturbances and sleep problems reported in young populations [53–55]. These correlational patterns validate the construct of “psychological distress” as a cohesive yet multifaceted phenomenon in our college student cohort. Second, the robust positive associations between psychological distress and both somatization and AIB underscore the psychosomatic interplay central to our study. Somatization showed strong correlations with depression and anxiety (*r* = 0.696 and *r* = 0.764, respectively), and a moderate correlation with insomnia (*r* = 0.540)—findings that echo prior research demonstrating that emotional distress frequently presents alongside somatic complaints (e.g., muscle tension, fatigue, headache, pain ) [46, 56–59]. Similarly, AIB was significantly correlated with all three distress indicators (*r* = 0.495 for anxiety, *r* = 0.533 for depression, *r* = 0.439 for insomnia), consistent with clinical observations that excessive health worry and maladaptive health-seeking behaviors tend to co-occur with unresolved psychological distress [13]. Notably, somatization and AIB also exhibited a moderate positive correlation (*r* = 0.576). This finding is consistent with recent evidence indicating that multifocal somatic symptoms are associated with AIB [24], and the present study further extends this associative pattern to young adults, a population in which such relationships have been rarely examined. Third, the negative correlations between psychological distress, somatization, AIB, and QoL components highlight the functional implications of these psychosomatic associations. Compared with the PCS, the MCS demonstrated a stronger negative correlation with various distress indicators (r range = -0.383 to -0.450 for MCS vs. -0.180 to -0.316 for PCS), which is consistent with existing research conclusions on the association between emotional distress and mental health-related quality of life, highlighting the necessity of distinguishing between mental and physical dimensions [60, 61]. Importantly, both AIB and somatization were moderately negatively correlated with MCS (*r* = -0.393 and *r* = -0.383, respectively)—though the magnitude was slightly weaker than that between psychological distress and MCS—indicating that beyond the observed association between psychological distress and MCS, AIB and somatization are also related to impaired mental health. Collectively, these correlational patterns not only confirm the theoretical plausibility of our moderated mediation model but also replicate and extend prior findings by demonstrating these interrelationships in a unified framework, laying the empirical groundwork for our subsequent moderated mediation analyses.

The moderating effect of somatization on the AIB-mediated pathway between psychological distress and quality of life was empirically corroborated by conditional process analysis, clarifying the long-debated interplay between somatization and AIB. As highlighted by Chaturvedi, somatization and AIB frequently co-occur [11, 62]; however, their interactive effects on functional outcomes—especially the regulatory patterns across different subtypes of psychological distress—remain poorly understood. By examining the dose-dependent manner through which somatization moderates the mediating role of AIB, this study further elucidates the dynamics of this psychosomatic pathway and provides more targeted empirical support for the field. Notably, conditional indirect effects showed a consistent pattern across all psychological distress subtypes and QoL domains. The index of moderated mediation with somatization as moderator was significant in all models: Anxiety → AIB → PCS (B = − 0.0009), Anxiety → AIB → MCS (B = − 0.0010), Depression → AIB → PCS (B = − 0.0008), Depression → AIB → MCS (B = − 0.0009), Insomnia → AIB → PCS (B = − 0.0007), and Insomnia → AIB → MCS (B = − 0.0012). These stable moderated mediation indices support the robustness and generalizability of the proposed psychosomatic models across different distress presentations and quality of life domains. Furthermore, the conditional indirect effect of AIB was significant at all levels of somatization, and the effect magnitude gradually strengthened as somatization intensified. Specifically, across low, moderate, and high somatization levels, conditional indirect effects were B = − 0.0192 to − 0.0312 (PCS) and B = − 0.0218 to − 0.0354 (MCS) for the anxiety pathways, B = − 0.0220 to − 0.0326 (PCS) and B = − 0.0250 to − 0.0371 (MCS) for the depression pathways, and B = − 0.0231 to − 0.0317 (PCS) and B = − 0.0423 to − 0.0580 (MCS) for the insomnia pathways. This pattern aligns well with the classic “signal amplifier” model proposed in previous studies [11, 13, 14, 63, 64]. This amplifying pattern indicates that students with higher somatization levels show stronger associations between AIB and impaired QoL, underscoring the need to prioritize somatization alongside AIB in targeted campus mental health interventions.

Taken together, the present study indicates that somatization and AIB act in a synergistic, amplifying manner rather than a merely concurrent relationship, which helps clarify the long-standing ambiguity regarding their interactive mechanisms in prior literature [11, 62]; despite notable overlap across psychological distress dimensions, the unified moderated mediation pattern observed across all subtypes reinforces the value of examining transdiagnostic psychosomatic processes while also supporting the need to understand their shared underlying correlates. Translating these findings into clinical and educational practice, our results offer localized implications for formulating targeted intervention programs for college students experiencing psychological distress. The core lies in adopting a “mind-body integration” orientation to precisely target two key nodes: somatization and AIB [63]. Interventions for this population should prioritize alleviating the severity of somatization symptoms—for instance, through psychological education to guide individuals in correctly interpreting somatic sensations associated with psychological distress, as the misinterpretation of such sensations plays a pivotal role in sustaining distress and functional impairment [65]. Meanwhile, cognitive-behavioral therapy can be culturally adapted and tailored to this group, focusing on correcting college students’ misinterpretations of somatic symptoms and catastrophic thinking to help them establish a comprehensive illness perception model [66]. In addition,, mind-body intervention techniques such as mindfulness meditation, resonant breathing, and Tai Chi can be incorporated to further reduce somatization symptoms by enhancing body awareness and stress management abilities [59, 66]. Considering the Chinese cultural context, somatization often serves as a socially acceptable, stigma-free medium for expressing psychological distress. Thus, clinical interventions need to explicitly validate individuals’ somatic experiences and reconstruct their negative illness cognitions through optimized doctor-patient communication, thereby effectively reducing the manifestation of AIB [8]. This aligns with previous research conclusions that early identification of multiple somatic symptoms helps reduce doctor-shopping behavior and excessive consumption of medical resources among individuals with AIB, ultimately improving the overall effectiveness of interventions [11].

In addition, the findings of this study have expanded the connotation of cross-cultural practical value and precision intervention, making them particularly applicable to non-Western cultural contexts. Based on the moderated mediation pattern of “psychological distress–somatization–AIB–QoL”, clinical practice should fully consider the role of somatization and adopt a graduated intervention strategy rather than a one-size-fits-all approach. Given that the mediating role of AIB increases progressively across the full spectrum of somatization severity, interventions should be flexibly adjusted according to somatization levels: the higher the somatization level, the more emphasis should be placed on interventions targeting abnormal illness behavior, while psychological distress counseling should be provided consistently across all severity levels. Integrating this graduated, symptom-adapted intervention logic into the existing mental health service system via efficient and convenient educational resources of colleges and universities is expected to effectively alleviate the impairment of quality of life among college students at a systemic level. Notably, since regions such as Southeast Asia also have high rates of mental health stigma and somatization [67, 68], this intervention framework demonstrates favorable cultural adaptability. It highlights the potential value of the study’s model in guiding global college student mental health practices and provides an important reference for constructing a student mental health service system rooted in localization and with an international vision.

Several limitations should be noted. First, the cross-sectional design of this study only allows for the identification of statistical associations among variables, and causal relationships cannot be inferred from the current results. Second, reliance on self-report questionnaires may introduce response biases. Third, the sample was restricted to Chinese college students, limiting generalizability to other age groups or cultural contexts. Future longitudinal studies with clinician-rated assessments, as well as cross-age and cross-cultural investigations, are needed to further validate these pathways and extend their generalizability.

## Conclusion

This study demonstrates that AIB mediates the relationship between psychological distress and QoL, and this mediation is moderated by somatization among Chinese college students. These findings clarify the psychosomatic correlates underlying QoL impairment in a non-Western population and provide a foundation for the development of culturally tailored college mental health strategies and clinical assessment protocols.

## Supplementary Information

Supplementary Material 1.

## Data Availability

Data is provided within the manuscript.
