# Supplementary material for: The extra burden: differential somatization-moderated mediation models of anxiety, depression, and insomnia on quality of life through abnormal illness behavior in Chinese college students
Source: BMC Psychol. 2026 Apr 18;14:970. doi: 10.1186/s40359-026-04461-1 (PMC13330162; doi:10.1186/s40359-026-04461-1)
Supplement: Supplementary file 1 — Supplementary Material 1. [file 40359_2026_4461_MOESM1_ESM.docx]

Supplementary Table 1. Univariate analysis was performed using linear regression within the generalized linear model, with PCS as the dependent variable.

| Variables | With PCS as the dependent variable | | | | |
| --- | --- | --- | --- | --- | --- |
|  | β | Standard error | Wald | 95% Confidence interval | P |
| Professional classification |  |  |  |  |  |
| Medical students |  |  |  |  | 1(REF) |
| Nonmedical students | -0.562 | 0.1369 | 16.852 | (-0.830,-0.294) | <0.001 |
| Gender |  |  |  |  |  |
| Male |  |  |  |  | 1(REF) |
| Female | 0.161 | 0.1607 | 1.002 | (-0.154,0.476) | 0.317 |
| Age | -0.083 | 0.0446 | 3.446 | (-0.17,0.005) | 0.063 |
| Geographical regions |  |  |  |  |  |
| East |  |  |  |  | 1(REF) |
| Central | -0.235 | 0.327 | 0.518 | (-0.876,0.405) | 0.472 |
| West | -0.181 | 0.3808 | 0.226 | (-0.927,0.565) | 0.635 |
| Northeast | -0.913 | 0.5863 | 2.427 | (-2.062,0.236) | 0.119 |
| Study duration |  |  |  |  |  |
| <6h |  |  |  |  | 1(REF) |
| ≥6h | 0.497 | 0.1368 | 13.212 | (0.229,0.766) | <0.001 |
| Exercise duration |  |  |  |  |  |
| <0.5h |  |  |  |  | 1(REF) |
| ≥0.5h | 0.624 | 0.1441 | 18.767 | (0.342,0.907) | <0.001 |
| Medical History |  |  |  |  |  |
| Mental illness | -1.975 | 0.387 | 26.056 | (-2.734,-1.217) | <0.001 |
| Medication use | -3.131 | 1.0173 | 9.476 | (-5.125,-1.138) | 0.002 |
| Hospitalization | -0.757 | 0.1783 | 18.048 | (-1.107,-0.408) | <0.001 |
| Allergies | -0.231 | 0.2112 | 1.194 | (-0.645,0.183) | 0.275 |
| Lifestyle factors |  |  |  |  |  |
| Alcohol drinking | -1.859 | 0.6384 | 8.474 | (-3.110,-0.607) | 0.004 |
| Cigarette smoking | -0.237 | 0.5382 | 0.194 | (-1.292,0.818) | 0.660 |
| Coffee or tea consumption | -0.757 | 0.1761 | 18.467 | (-1.102,-0.412) | <0.001 |
| Multi-dimensional health perception | 0.941 | 0.1361 | 47.836 | (0.674,1.208) | <0.001 |
| Total stress | -0.419 | 0.0432 | 94.361 | (-0.504,-0.335) | <0.001 |
| Well-being | 0.716 | 0.0536 | 178.627 | (0.611,0.821) | <0.001 |
| Abnormal illness beahavior | -0.982 | 0.0499 | 387.146 | (-1.079,-0.884) | <0.001 |
| Anxiety | -0.295 | 0.0207 | 202.818 | (-0.335,-0.254) | <0.001 |
| Depression | -0.300 | 0.0182 | 271.589 | (-0.336,-0.265) | <0.001 |
| Insomnia | -0.470 | 0.0192 | 598.411 | (-0.508,-0.432) | <0.001 |
| Somatizaiton | -0.206 | 0.0099 | 428.066 | (-0.225,-0.186) | <0.001 |

REF, reference.

Supplementary Table 2. Univariate analysis was performed using linear regression within the generalized linear model, with MCS as the dependent variable.

| Variables | With MCS as the dependent variable | | | | |
| --- | --- | --- | --- | --- | --- |
|  | β | Standard error | Wald | 95% Confidence interval | P |
| Professional classification |  |  |  |  |  |
| Medical students |  |  |  |  | 1(REF) |
| Nonmedical students | -0.475 | 0.1796 | 6.989 | (-0.827,-0.123) | 0.008 |
| Gender |  |  |  |  |  |
| Male |  |  |  |  | 1(REF) |
| Female | 1.853 | 0.218 | 72.307 | (1.426,2.281) | <0.001 |
| Age | -0.186 | 0.0588 | 10.022 | (-0.301,-0.071) | 0.002 |
| Geographical regions |  |  |  |  |  |
| East |  |  |  |  | 1(REF) |
| Central | 0.291 | 0.4245 | 0.471 | (-0.541,1.123) | 0.492 |
| West | -0.584 | 0.502 | 1.352 | (-1.568,0.400) | 0.245 |
| Northeast | 0.771 | 0.7713 | 0.998 | (-0.741,2.282) | 0.318 |
| Study duration |  |  |  |  |  |
| <6h |  |  |  |  | 1(REF) |
| ≥6h | 1.318 | 0.1799 | 53.635 | (0.965,1.670) | <0.001 |
| Exercise duration |  |  |  |  |  |
| <0.5h |  |  |  |  | 1(REF) |
| ≥0.5h | 1.426 | 0.1858 | 58.852 | (1.061,1.790) | <0.001 |
| Medical History |  |  |  |  |  |
| Mental illness | -6.164 | 0.4862 | 160.75 | (-7.117,-5.211) | <0.001 |
| Medication use | -6.356 | 1.1435 | 30.899 | (-8.597,-4.115) | <0.001 |
| Hospitalization | -1.35 | 0.2344 | 33.179 | (-1.810,-0.891) | <0.001 |
| Allergies | 0.092 | 0.2712 | 0.115 | (-0.440,0.623) | 0.735 |
| Lifestyle factors |  |  |  |  |  |
| Alcohol drinking | -4.931 | 0.7681 | 41.207 | (-6.437,-3.425) | <0.001 |
| Cigarette smoking | -2.572 | 0.7483 | 11.813 | (-4.038,-1.105) | 0.001 |
| Coffee or tea consumption | -1.417 | 0.2292 | 38.204 | (-1.866,-0.968) | <0.001 |
| Multi-dimensional health perception | 1.816 | 0.1796 | 102.305 | (1.464,2.168) | <0.001 |
| Total stress | -1.167 | 0.0625 | 348.277 | (-1.290,-1.044) | <0.001 |
| Well-being | 2.462 | 0.0632 | 1519.709 | (2.338,2.586) | <0.001 |
| Abnormal illness beahavior | -2.04 | 0.0575 | 1258.475 | (-2.153,-1.928) | <0.001 |
| Anxiety | -0.977 | 0.0241 | 1642.344 | (-1.024,-0.930) | <0.001 |
| Depression | -0.874 | 0.0207 | 1782.236 | (-0.914,-0.833) | <0.001 |
| Insomnia | -0.87 | 0.024 | 1317.745 | (-0.917,-0.823) | <0.001 |
| Somatizaiton | -0.456 | 0.0117 | 1526.165 | (-0.479,-0.433) | <0.001 |

REF, reference.

Supplementary Table 3. Multicollinearity diagnosis: Multiple linear regression analysis with PCS as the dependent variable

| Variables | With PCS as the dependent variable | | | | | | | | |
| --- | --- | --- | --- | --- | --- | --- | --- | --- | --- |
|  | β | SE | Std.β | t | P | Tolerance | VIF | Eigenvalue | CI |
| Anxiety Model (R^2^ = 0.083, F = 4.793 , DW = 1.864, P < 0.001 ) | | | | | | | | | |
| Constant | 55.633 | 3.459 |  | 16.083 | 0.000 | - | - | 10.836 | 1.000 |
| Professional classification | -1.085 | 0.439 | -0.085 | -2.473 | 0.014 | 0.853 | 1.172 | 1.366 | 2.816 |
| Age | -0.144 | 0.140 | -0.035 | -1.027 | 0.305 | 0.862 | 1.160 | 1.163 | 3.052 |
| Gender | -0.505 | 0.472 | -0.036 | -1.069 | 0.285 | 0.893 | 1.120 | 0.732 | 3.847 |
| Study duarion | -0.198 | 0.433 | -0.015 | -0.458 | 0.647 | 0.931 | 1.075 | 0.703 | 3.926 |
| Exercise duration | -0.012 | 0.438 | -0.001 | -0.027 | 0.978 | 0.867 | 1.154 | 0.672 | 4.014 |
| Mental illness | 0.178 | 0.669 | 0.009 | 0.266 | 0.790 | 0.868 | 1.152 | 0.617 | 4.192 |
| Medication use | -1.854 | 1.546 | -0.040 | -1.200 | 0.231 | 0.899 | 1.112 | 0.574 | 4.346 |
| Hospitalization | -0.396 | 0.470 | -0.027 | -0.841 | 0.401 | 0.950 | 1.053 | 0.421 | 5.072 |
| Alcohol drinking | 0.242 | 1.138 | 0.007 | 0.213 | 0.831 | 0.846 | 1.182 | 0.288 | 6.137 |
| Cigarette smoking | 0.431 | 1.215 | 0.013 | 0.354 | 0.723 | 0.807 | 1.239 | 0.179 | 7.787 |
| Coffee or tea consumption | 0.500 | 0.458 | 0.035 | 1.092 | 0.275 | 0.960 | 1.041 | 0.142 | 8.739 |
| Somatizaiton | -0.166 | 0.040 | -0.162 | -4.134 | 0.000 | 0.660 | 1.515 | 0.109 | 9.993 |
| Total stress | 0.048 | 0.120 | 0.014 | 0.397 | 0.691 | 0.860 | 1.162 | 0.088 | 11.112 |
| Well-being | 0.153 | 0.149 | 0.036 | 1.027 | 0.304 | 0.819 | 1.221 | 0.063 | 13.138 |
| Abnormal illness beahavior | -0.511 | 0.120 | -0.153 | -4.265 | 0.000 | 0.791 | 1.263 | 0.035 | 17.614 |
| Multi-dimensional health perception | 0.877 | 0.440 | 0.065 | 1.995 | 0.046 | 0.944 | 1.060 | 0.010 | 32.451 |
| Anxiety | 0.232 | 0.059 | 0.148 | 3.950 | 0.000 | 0.721 | 1.387 | 0.002 | 69.354 |
| Depression Model (R^2^ =0.076 , F = 4.374 , DW = 1.867, P < 0.001 ) | | | | | | | | | |
| Constant | 55.951 | 3.476 |  | 16.095 | 0.000 | - | - | 10.866 | 1.000 |
| Professional classification | -1.045 | 0.440 | -0.082 | -2.375 | 0.018 | 0.854 | 1.171 | 1.366 | 2.820 |
| Age | -0.157 | 0.141 | -0.038 | -1.114 | 0.266 | 0.863 | 1.159 | 1.163 | 3.057 |
| Gender | -0.424 | 0.473 | -0.030 | -0.895 | 0.371 | 0.895 | 1.118 | 0.732 | 3.854 |
| Study duarion | -0.092 | 0.434 | -0.007 | -0.211 | 0.833 | 0.930 | 1.076 | 0.703 | 3.931 |
| Exercise duration | -0.032 | 0.439 | -0.003 | -0.073 | 0.942 | 0.867 | 1.154 | 0.672 | 4.022 |
| Mental illness | 0.177 | 0.673 | 0.009 | 0.263 | 0.793 | 0.863 | 1.158 | 0.617 | 4.195 |
| Medication use | -1.600 | 1.551 | -0.035 | -1.032 | 0.302 | 0.900 | 1.111 | 0.578 | 4.335 |
| Hospitalization | -0.423 | 0.472 | -0.029 | -0.895 | 0.371 | 0.950 | 1.052 | 0.417 | 5.105 |
| Alcohol drinking | 0.347 | 1.142 | 0.011 | 0.303 | 0.762 | 0.846 | 1.181 | 0.286 | 6.167 |
| Cigarette smoking | 0.557 | 1.219 | 0.016 | 0.457 | 0.648 | 0.808 | 1.237 | 0.177 | 7.846 |
| Coffee or tea consumption | 0.466 | 0.460 | 0.033 | 1.013 | 0.311 | 0.959 | 1.043 | 0.121 | 9.487 |
| Somatizaiton | -0.168 | 0.043 | -0.163 | -3.936 | 0.000 | 0.593 | 1.685 | 0.107 | 10.085 |
| Total stress | 0.027 | 0.121 | 0.008 | 0.226 | 0.821 | 0.859 | 1.164 | 0.089 | 11.043 |
| Well-being | 0.123 | 0.149 | 0.029 | 0.824 | 0.410 | 0.820 | 1.219 | 0.063 | 13.166 |
| Abnormal illness beahavior | -0.496 | 0.120 | -0.148 | -4.131 | 0.000 | 0.793 | 1.261 | 0.032 | 18.476 |
| Multi-dimensional health perception | 0.930 | 0.441 | 0.069 | 2.108 | 0.035 | 0.945 | 1.059 | 0.010 | 33.466 |
| Depression | 0.173 | 0.058 | 0.121 | 2.995 | 0.003 | 0.624 | 1.603 | 0.002 | 69.556 |
| Insomnia Model (R^2^ = 0.075 , F = 4.349 , DW = 1.884, P < 0.001 ) | | | | | | | | | |
| Constant | 54.696 | 3.479 |  | 15.720 | 0.000 | - | - | 10.824 | 1.000 |
| Professional classification | -1.033 | 0.440 | -0.081 | -2.347 | 0.019 | 0.854 | 1.171 | 1.366 | 2.815 |
| Age | -0.132 | 0.141 | -0.032 | -0.932 | 0.351 | 0.859 | 1.164 | 1.163 | 3.050 |
| Gender | -0.456 | 0.473 | -0.033 | -0.962 | 0.336 | 0.894 | 1.118 | 0.731 | 3.849 |
| Study duarion | -0.179 | 0.434 | -0.014 | -0.413 | 0.680 | 0.931 | 1.074 | 0.704 | 3.922 |
| Exercise duration | -0.151 | 0.440 | -0.012 | -0.343 | 0.732 | 0.866 | 1.154 | 0.672 | 4.014 |
| Mental illness | 0.585 | 0.672 | 0.030 | 0.870 | 0.385 | 0.866 | 1.155 | 0.618 | 4.186 |
| Medication use | -1.523 | 1.551 | -0.033 | -0.982 | 0.326 | 0.900 | 1.111 | 0.578 | 4.326 |
| Hospitalization | -0.446 | 0.472 | -0.031 | -0.946 | 0.345 | 0.951 | 1.051 | 0.418 | 5.087 |
| Alcohol drinking | 0.868 | 1.140 | 0.026 | 0.761 | 0.447 | 0.850 | 1.177 | 0.294 | 6.067 |
| Cigarette smoking | 0.593 | 1.219 | 0.017 | 0.486 | 0.627 | 0.808 | 1.237 | 0.172 | 7.924 |
| Coffee or tea consumption | 0.584 | 0.460 | 0.041 | 1.269 | 0.205 | 0.959 | 1.043 | 0.149 | 8.515 |
| Somatizaiton | -0.074 | 0.039 | -0.072 | -1.906 | 0.057 | 0.705 | 1.418 | 0.107 | 10.047 |
| Total stress | 0.068 | 0.121 | 0.019 | 0.560 | 0.576 | 0.856 | 1.168 | 0.090 | 10.963 |
| Well-being | 0.006 | 0.147 | 0.001 | 0.040 | 0.968 | 0.847 | 1.180 | 0.064 | 13.002 |
| Abnormal illness beahavior | -0.423 | 0.122 | -0.126 | -3.478 | 0.001 | 0.774 | 1.292 | 0.036 | 17.374 |
| Multi-dimensional health perception | 0.907 | 0.441 | 0.068 | 2.055 | 0.040 | 0.944 | 1.059 | 0.011 | 31.578 |
| Insomnia | -0.150 | 0.051 | -0.107 | -2.926 | 0.004 | 0.764 | 1.309 | 0.002 | 69.534 |

CI: Condition Index. For variables with a maximum CI > 30, the variance proportions of multiple independent variables under any CI dimension did not reach ≥ 0.5.

Supplementary Table 4. Multicollinearity diagnosis: Multiple linear regression analysis with MCS as the dependent variable

| Variables | With MCS as the dependent variable | | | | | | | | |
| --- | --- | --- | --- | --- | --- | --- | --- | --- | --- |
|  | β | SE | Std.β | t | P | Tolerance | VIF | Eigenvalue | CI |
| Anxiety Model (R^2^ = 0.255 , F = 18.272 , DW = 1.946, P < 0.001 ) | | | | | | | | | |
| Constant | 47.308 | 3.371 |  | 14.034 | 0.000 | - |  | 10.836 | 1.000 |
| Professional classification | 0.408 | 0.428 | 0.030 | 0.953 | 0.341 | 0.853 | 1.172 | 1.366 | 2.816 |
| Age | -0.089 | 0.137 | -0.020 | -0.650 | 0.516 | 0.862 | 1.160 | 1.163 | 3.052 |
| Gender | 1.283 | 0.460 | 0.085 | 2.789 | 0.005 | 0.893 | 1.120 | 0.732 | 3.847 |
| Study duarion | 0.796 | 0.422 | 0.056 | 1.888 | 0.059 | 0.931 | 1.075 | 0.703 | 3.926 |
| Exercise duration | 0.054 | 0.427 | 0.004 | 0.126 | 0.900 | 0.867 | 1.154 | 0.672 | 4.014 |
| Mental illness | -1.804 | 0.652 | -0.085 | -2.767 | 0.006 | 0.868 | 1.152 | 0.617 | 4.192 |
| Medication use | 0.841 | 1.506 | 0.017 | 0.558 | 0.577 | 0.899 | 1.112 | 0.574 | 4.346 |
| Hospitalization | 0.436 | 0.458 | 0.028 | 0.952 | 0.341 | 0.950 | 1.053 | 0.421 | 5.072 |
| Alcohol drinking | -0.650 | 1.109 | -0.018 | -0.586 | 0.558 | 0.846 | 1.182 | 0.288 | 6.137 |
| Cigarette smoking | 0.797 | 1.184 | 0.021 | 0.673 | 0.501 | 0.807 | 1.239 | 0.179 | 7.787 |
| Coffee or tea consumption | -0.316 | 0.446 | -0.021 | -0.708 | 0.479 | 0.960 | 1.041 | 0.142 | 8.739 |
| Somatizaiton | -0.043 | 0.039 | -0.039 | -1.110 | 0.267 | 0.660 | 1.515 | 0.109 | 9.993 |
| Total stress | -0.259 | 0.117 | -0.068 | -2.214 | 0.027 | 0.860 | 1.162 | 0.088 | 11.112 |
| Well-being | 0.844 | 0.145 | 0.184 | 5.823 | 0.000 | 0.819 | 1.221 | 0.063 | 13.138 |
| Abnormal illness beahavior | -0.592 | 0.117 | -0.163 | -5.064 | 0.000 | 0.791 | 1.263 | 0.035 | 17.614 |
| Multi-dimensional health perception | 0.529 | 0.428 | 0.036 | 1.235 | 0.217 | 0.944 | 1.060 | 0.010 | 32.451 |
| Anxiety | -0.420 | 0.057 | -0.247 | -7.329 | 0.000 | 0.721 | 1.387 | 0.002 | 69.354 |
| Depression Model (R^2^ = 0.266 , F = 19.332 , DW = 1.931, P < 0.001 ) | | | | | | | | | |
| Constant | 46.246 | 3.351 |  | 13.801 | 0.000 | - |  | 10.866 | 1.000 |
| Professional classification | 0.345 | 0.424 | 0.025 | 0.813 | 0.416 | 0.854 | 1.171 | 1.366 | 2.820 |
| Age | -0.068 | 0.136 | -0.015 | -0.498 | 0.619 | 0.863 | 1.159 | 1.163 | 3.057 |
| Gender | 1.140 | 0.456 | 0.075 | 2.500 | 0.013 | 0.895 | 1.118 | 0.732 | 3.854 |
| Study duarion | 0.557 | 0.419 | 0.039 | 1.330 | 0.184 | 0.930 | 1.076 | 0.703 | 3.931 |
| Exercise duration | 0.044 | 0.423 | 0.003 | 0.103 | 0.918 | 0.867 | 1.154 | 0.672 | 4.022 |
| Mental illness | -1.615 | 0.649 | -0.076 | -2.489 | 0.013 | 0.863 | 1.158 | 0.617 | 4.195 |
| Medication use | 0.340 | 1.495 | 0.007 | 0.227 | 0.820 | 0.900 | 1.111 | 0.578 | 4.335 |
| Hospitalization | 0.446 | 0.455 | 0.029 | 0.980 | 0.327 | 0.950 | 1.052 | 0.417 | 5.105 |
| Alcohol drinking | -0.579 | 1.101 | -0.016 | -0.526 | 0.599 | 0.846 | 1.181 | 0.286 | 6.167 |
| Cigarette smoking | 0.592 | 1.175 | 0.016 | 0.504 | 0.615 | 0.808 | 1.237 | 0.177 | 7.846 |
| Coffee or tea consumption | -0.205 | 0.443 | -0.013 | -0.463 | 0.643 | 0.959 | 1.043 | 0.121 | 9.487 |
| Somatizaiton | 0.010 | 0.041 | 0.009 | 0.250 | 0.803 | 0.593 | 1.685 | 0.107 | 10.085 |
| Total stress | -0.209 | 0.116 | -0.055 | -1.797 | 0.073 | 0.859 | 1.164 | 0.089 | 11.043 |
| Well-being | 0.826 | 0.144 | 0.181 | 5.746 | 0.000 | 0.820 | 1.219 | 0.063 | 13.166 |
| Abnormal illness beahavior | -0.605 | 0.116 | -0.167 | -5.224 | 0.000 | 0.793 | 1.261 | 0.032 | 18.476 |
| Multi-dimensional health perception | 0.436 | 0.425 | 0.030 | 1.024 | 0.306 | 0.945 | 1.059 | 0.010 | 33.466 |
| Depression | -0.460 | 0.056 | -0.297 | -8.242 | 0.000 | 0.624 | 1.603 | 0.002 | 69.556 |
| Insomnia Model (R^2^ = 0.241 , F = 16.916 , DW = 1.931, P < 0.001 ) | | | | | | | | | |
| Constant | 46.419 | 3.410 |  | 13.611 | 0.000 | - | - | 10.824 | 1.000 |
| Professional classification | 0.322 | 0.432 | 0.023 | 0.746 | 0.456 | 0.854 | 1.171 | 1.366 | 2.815 |
| Age | -0.009 | 0.138 | -0.002 | -0.063 | 0.950 | 0.859 | 1.164 | 1.163 | 3.050 |
| Gender | 1.055 | 0.464 | 0.070 | 2.274 | 0.023 | 0.894 | 1.118 | 0.731 | 3.849 |
| Study duarion | 0.638 | 0.426 | 0.045 | 1.498 | 0.135 | 0.931 | 1.074 | 0.704 | 3.922 |
| Exercise duration | 0.062 | 0.431 | 0.004 | 0.144 | 0.885 | 0.866 | 1.154 | 0.672 | 4.014 |
| Mental illness | -1.826 | 0.659 | -0.086 | -2.772 | 0.006 | 0.866 | 1.155 | 0.618 | 4.186 |
| Medication use | 0.724 | 1.521 | 0.015 | 0.476 | 0.634 | 0.900 | 1.111 | 0.578 | 4.326 |
| Hospitalization | 0.614 | 0.462 | 0.039 | 1.328 | 0.185 | 0.951 | 1.051 | 0.418 | 5.087 |
| Alcohol drinking | -0.966 | 1.118 | -0.027 | -0.864 | 0.388 | 0.850 | 1.177 | 0.294 | 6.067 |
| Cigarette smoking | 0.539 | 1.195 | 0.015 | 0.451 | 0.652 | 0.808 | 1.237 | 0.172 | 7.924 |
| Coffee or tea consumption | -0.240 | 0.451 | -0.016 | -0.533 | 0.594 | 0.959 | 1.043 | 0.149 | 8.515 |
| Somatizaiton | -0.086 | 0.038 | -0.077 | -2.246 | 0.025 | 0.705 | 1.418 | 0.107 | 10.047 |
| Total stress | -0.201 | 0.118 | -0.053 | -1.701 | 0.089 | 0.856 | 1.168 | 0.090 | 10.963 |
| Well-being | 0.990 | 0.144 | 0.217 | 6.885 | 0.000 | 0.847 | 1.180 | 0.064 | 13.002 |
| Abnormal illness beahavior | -0.532 | 0.119 | -0.147 | -4.464 | 0.000 | 0.774 | 1.292 | 0.036 | 17.374 |
| Multi-dimensional health perception | 0.380 | 0.432 | 0.026 | 0.878 | 0.380 | 0.944 | 1.059 | 0.011 | 31.578 |
| Insomnia | -0.300 | 0.050 | -0.197 | -5.961 | 0.000 | 0.764 | 1.309 | 0.002 | 69.534 |

CI: Condition Index. For variables with a maximum CI > 30, the variance proportions of multiple independent variables under any CI dimension did not reach ≥ 0.5.
